# Associations Between Depressive Symptoms and Physical Activity Intensity in an Older Adult Population During COVID-19 Lockdown

**DOI:** 10.3389/fpsyg.2021.644106

**Published:** 2021-06-07

**Authors:** Ana Lage, Susana Carrapatoso, Elzier Sampaio de Queiroz Neto, Sérgio Gomes, Luísa Soares-Miranda, Lucimere Bohn

**Affiliations:** ^1^Research Center in Physical Activity, Health and Leisure (CIAFEL), Faculty of Sports, University of Porto (FADEUP), Portugal Laboratory for Integrative and Translational Research in Population Health (ITR), Porto, Portugal; ^2^Faculty of Phycology, Education and Sport, University Lusófona of Porto, Porto, Portugal; ^3^Prefeitura Municipal de Fortaleza, Coordenadoria do Idoso, Fortaleza, Brazil; ^4^Department of Hematology, Centro Hospitalar Universitário do Porto, Porto, Portugal

**Keywords:** depression, physical activity, pandemic, lockdown, association, intensity

## Abstract

**Introduction:**

The COVID-19 pandemic led to the implementation of physical–social distancing measures–including self-isolation, home confinement, and quarantine around the world, with psychological consequences such as depression. Older adults are especially likely to develop depressive symptomatology. This study aims to investigate the association between physical activity intensities and sedentary behavior with depression levels among previously active older adults during the COVID-19 lockdown.

**Methods:**

A total of 1,123 physically active older Brazilian adults (67.68 ± 5.91 years, 91.00% female) were interviewed by telephone in regard to sociodemographic, general health status, depression (GDS-15), and physical activity (IPAQ-SV) after being home-confined for 11.59 ± 2.42 weeks. Participants were also asked to self-report changes in their physical activity levels and time spent sitting. Descriptive statistics (mean, frequencies), between-groups comparisons (*t*-tests and chi-square), and hierarchical regression analysis were used.

**Results:**

About 83.80% of older adults self-reported a decrease in daily physical activity levels and 73.90% increased sitting time. Overall, depressive symptoms were observed in 30.40, and 20.80% met physical activity recommendations. Daily moderate (β = −0.174; 95% CI = −0.026; -0.012) and moderate-to-vigorous (β = −0.183; 95% CI = −0.023; 0.011) physical activity intensities were negatively associated with depression score explaining 2.6 and 2.9% of depression variability, respectively, after adjusting for age, gender, education level, body mass index, and polypharmacy. Daily walking and sitting time were not associated with the depression score (*p* > 0.05).

**Conclusion:**

The results provide empiric suggestion supporting moderate to vigorous physical activity as a way to reduce depressive levels among older adults during COVID-19 confinement. Supervised home-based exercise programs, specifically designed for older adults, might be an important strategy to maintain and improve older adults’ mental health.

## Introduction

The world is facing the devastating effects of the COVID-19 outbreak, an infection caused by the virus SARS-CoV2 that rapidly propagated within communities. To contain the spread of the virus and to protect the most fragile groups, several countries have implemented strict measures of physical–social distancing, such as self-isolation, home confinement, and quarantine (Armitage and Nellums, 2020). These measures are indeed efficient to mitigate virus activity, with epidemiological data demonstrating an abrupt reduction in the number of new COVID-19 cases and consequently mortality (Shahid et al., 2020). Nevertheless, these measures are not free from psychological detrimental side effects, as augmented loneliness may have adverse effects in psychological distress among the general population (Banerjee and Rai, 2020), especially in the most vulnerable groups as older adults. Santini et al. (2020) have already demonstrated that the social isolation and consequent loneliness during the outbreak have increased depression symptoms such as persistent low mood, dysphoria, and impaired motivation in older adults. Considering that the prevalence of depression among older adults in Western countries is around 20% (Volkert et al., 2013), an important worsening of this health outcome as a result of this sanitary crisis can be expected (Armitage and Nellums, 2020).

Social isolation as a result of home confinement also affected daily physical activity levels and sedentariness behavior in older adults, since the possibilities to perform physical exercise were reduced, and activities in the sitting or reclined position (watching television, reading, crocheting, and knitting, etc.) were exponentially increased, as recent available evidence indicates (Schrempft et al., 2019; Jakobsson et al., 2020; Jiménez-Pavón et al., 2020).

The World Health Organization (WHO) has recognized that social distancing, along with an excessive time spent sitting and a lower level of physical activity, might enlarge depressive symptoms within populations (Bull et al., 2020). Both sedentary behavior and physical inactivity negatively affect mood and are also associated with depressive symptoms (Mura and Carta, 2013; Mumba et al., 2020). In this sense, it has been suggested that physical activity could have an important role in the mental health promotion (and preservation) due to its potential to buffer adverse effects of stress (Roux, 2007). Empirical evidence has reported that a greater amount of total physical activity was associated with lower depressive symptoms (Callow et al., 2020). Physical exercise, a subcategory of physical activity, protects older adults from depression due to its positive effects on biological and psychological processes (social support, self-esteem, and the social relations inherent to exercise participation) (Perez-Lopez et al., 2017; Kandola et al., 2019). Moreover, a poor functional fitness status is also one of the most important causes of late-life depressive symptoms (Blazer and Hybels, 2005), which could be reverted through the participation in well-designed individual-tailored exercise programs (Bull et al., 2020). To that extent, it is expected that older adults who participated in physical exercise programs might be more protected against depressive symptoms caused by more restrictive measures such as home confinement. However, to the best of our knowledge, studies exploring the association between physical activity levels and depressive symptoms during social isolation imposed by COVID-19 outbreak in a population of active older adults are scarce. Therefore, the aim of the present study is to determine the relationship between intensities of physical activity performed by physically active older Brazilian adults and their depressive symptoms during home confinement.

## Materials and Methods

### Study Design

This is a cross-sectional observational study based in a non-probabilistic sample comprising 1,123 older Brazilian’s adults enrolled in the exercise program “Fortaleza Cidade Amiga do Idoso.” The exercise program is hosted at the “Núcleo de Produções Culturais e Esportivas” (NUPROCE), a non-governmental organization in Fortaleza, Ceará, Brazil. Eligibility criteria were physically active subjects aged ≥60 enrolled in the exercise program in the school year 2019–2020.

### Participants and Procedures

“Fortaleza Cidade Amiga do Idoso” consists of an in-person social program that offers social, cultural, and sports activities. The latter include multicomponent training, hydro gymnastics, dance, and walking–running sessions. Older adults are free to choose the activities they want to enroll, as well as the weekly frequency. Each session lasted 60 min and, prior to the outbreak, used to take place in public facilities. All older adults attending any sports activity from the program in the school year 2019–2020 were contacted by telephone and invited to participate in the study during home confinement. In Fortaleza, the lockdown was initiated on March 19th, and the reopening phase, which finished with the duty of confinement, started on July 12th. Along this period, people from Fortaleza were only allowed to get out from their homes to perform essential activities such as acquisition of goods and services and health reasons. Data were collected during the month of June (1st–31st) via phone calls, when subjects were still under home confinement. Participants’ contacts were provided by the NUPROCE secretariat after the study was approved by the ethics board.

#### Ethics Statement

The study was approved by the NUPROCE and the Secretaria de Direitos Humanos e Desenvolvimento Social of Fortaleza, Ceará, Brazil. All procedures followed the Helsinki Declaration. Each participant provided a verbal informed consent before starting to be interviewed. Participants could withdraw from the survey at any point in time without providing justification.

### Data Collection

Twenty interviewers, who used to lead older adults’ activities prior the confinement, were trained by three researchers to perform the interviews. The data were collected via telephone interviews, and answers were entered into a Google Form database. For each individual, there were up to six calling attempts, performed in different days and hours. As soon as the interviewers introduced a set of answers into the dataset, all three researchers checked for missing data or typing error.

#### Sociodemographic and Health-Related Conditions

Data on sociodemographic (sex, age, and years of education), lockdown duration, the number of exercise sessions per week previous to the lockdown, and the use of multiple medicines (i.e., polypharmacy) were obtained with open-answer questions. The presence of health conditions (hypertension, dyslipidemia, diabetes, cardiovascular disease, chronic respiratory disease, cancer, and COVID-19) was asked as yes/no questions.

#### Anthropometry

Body weight (kg) and height (meters) were self-reported, and the body mass index (BMI) was derived as body weight divided by squared height (kg/m^2^).

#### Symptoms of Depression

Symptoms of depression were determined using the Brazilian validated Geriatric Depression Scale–Short Form (GDS-15) (Almeida and Almeida, 1999a). The GDS-15 comprises of 15 questions examining the older adult’s mood in the previous week. Answers are “yes” or “no,” and 1 point is given either to the answer “yes” or to the answer “no” depending on the question. The final score is the sum of total answers, with higher results reflecting worse depressive states. Scores were coded ranging from no depression symptoms (0–4) and presence of depression symptoms (≥5) (Almeida and Almeida, 1999a). The validated Brazilian version presented a good internal consistency (Almeida and Almeida, 1999b).

#### Physical Activity

Physical activity was measured using the Brazilian validated short-version of the International Physical Activity Questionnaire (IPAQ-SV) (Craig et al., 2003). The participants were asked to provide information on frequency (days per week) and duration (hours or minutes per day) they spent walking, as well as in moderate and vigorous physical activity. The IPAQ-SV also includes a question about sitting time (time per day in sitting position). The daily duration of each intensity of physical activity (moderate and vigorous) and walking (i.e., light physical activity) was computed multiplying the number of days per week by the time per day in each intensity. After that, the results for each physical activity intensity and walking were divided by 7 to get the mean values per day (i.e., light physical activity/day, moderate physical activity/day, and vigorous physical activity/day). Additionally, moderate and vigorous physical activity per day was summed to get moderate to vigorous physical activity. Participants were also classified against physical activity guidelines (“<150 min per week” or “≥150 min per week” of moderate to vigorous physical activity) (Bull et al., 2020). Participants were asked to self-report changes in physical activity and time spent sitting during home confinement in comparison to the prior-confinement period. Answer options were reduced, maintained, and augmented.

### Statistical Analysis

Descriptive statistics and frequency analysis were used to describe the sample. Between-groups comparisons (no depression symptoms versus presence of depression symptoms) were performed using independent *t*-test and chi-square (χ^2^) test as appropriate. Hierarchical multiple linear regression analysis, with the enter method, was used to determine the unique contribution of each physical activity intensity variable and sitting time in predicting the depression score. The base model (model 1) included the factors that have plausibility to determine depression (age, gender, educational level, body mass index, and polypharmacy). Model 2 included daily walking (i.e., light physical activity) plus variables from model 1. Model 3 was computed based on model 2 plus moderate physical activity intensity. Model 4 included additional vigorous physical activity plus variables from model 3. Model 5 was computed in line with model 2 with moderate to vigorous physical activity as one single variable. Model 6 was equal to model 1 plus sitting time. Finally, model 7 included model 1 plus daily walking, moderate to vigorous physical activity, and sitting time. The homoscedasticity of the models (residual variance) was checked by visual inspection of dispersion between residues and predicted values. Multicollinearity was checked and considered acceptable when the variance inflation factor (VIF) <10 (Myers, 1990). Sensitivity analysis, excluding older male adults, was also performed for hierarchical regression. All procedures were carried out using the statistical package IBM SPSS Statistics software, version 26 (Chicago, United States). *p*-values <0.05 were considered significant.

## Results

One thousand four hundred fifty-three older adults enrolled in the exercise program were contacted. From those, 107 older adults had contact problems, 197 did not answer phone calls, and 11 declined to participate. In total, 1,123 agreed to participate (67.68 ± 5.91 years old, 91.00% females).

Overall sample characteristics and between-groups comparisons are depicted in Table 1. Older adults were subject to 11.59 ± 2.42 weeks of home confinement. The two lowest levels of education were the most frequent among the older adults (<6 years: 38.6%; 6–12 years: 54.3%), with significant differences in the distribution of education according to the presence or absence of depression symptoms (*p* = 0.008). Overweight and obesity were present in 47.2% on older adults, with significant BMI differences between groups (presence of depression symptoms: 27.49 ± 4.37 kg/m^2^; no depression symptoms: 26.92 ± 3.83 kg/m^2^; *p* = 0.040). Regarding the overall risk factors, 62.2% had hypertension and 44.90% had dyslipidemia. At the moment of data collection, 4.7% were infected with COVID-19, and 30.40% had presence of depression symptoms. On average, older adults spent 325.51 ± 144.40 min/day in sitting time during the confinement period. Regardless of the presence of depression symptoms, all older adults reported to have augmented their sitting time per day (73.90%), but it was more frequent within the group with depressive symptoms compared to the group without symptoms (83.30 versus 69.80%, respectively; *p* < 0.001). The depressive symptoms group spent, on average, 12.78 ± 25.73 min/day in moderate to vigorous physical activity, which was significantly lower compared to the mean of the group without depression symptoms (21.28 ± 31.02 min/day; *p* < 0.001). Overall, 83.80% of older adults diminished their physical activity levels, which was significantly higher within the group with depressive symptoms (92.10%) compared to the one without depressive symptoms (80.2%; *p* < 0.001); 20.8% of older adults met physical activity guidelines, with significant differences between groups (without depressive symptoms: 15%; depressive symptoms group: 23.5%; *p* < 0.001).

**TABLE 1:** Descriptive and between-groups comparisons.

Variables	Overall	No depression symptoms	Presence of depression symptoms	Statistical inference
	(*N*: 1123)	(*N*: 782)	(*N*: 341)	
**Age, years**	67.68 ± 5.91	67.66 ± 5.95	67.73 ± 5.87	*t* (1121) = −0.184; *p* = 0.854
**Gender**				
Female, %	91.00%	90.50%	91.20%	χ^2^(1, *N* = 1123) = 0.125; *p* = 0.409
Male, %	9.00%	9.50%	8.80%	
**Education**				
<6 years, %	38.6%	38.00%	39.90%	χ^2^: (2, *N* = 1123) = 9.624; *p* = 0.008
6–12 years, %	54.3%	53.30%	56.60%	
>12 years, %	7.10%	8.70%	3.50%	
**Anthropometry**				
Body mass index, kg/m^2^	27.08 ± 4.00	26.92 ± 3.83	27.49 ± 4.37	*t* (557.996) = -2.062; *p* = 0.040
Overweight and Obesity, *N* (%)	530 (47.20%)	359 (46.60%)	161 (48.60%)	χ^2^: (2, *N* = 1101) = 0.653; *p* = 0.721
**Health conditions**				
Hypertension, %	62.20%	58.80%	69.80%	χ^2^ (2, *N* = 1123) = 33.098; *p* < 0.001
Dyslipidemia, %	44.90%	42.80%	49.60%	χ^2^ (2, *N* = 1123) = 4.383; *p* = 0.112
Diabetes, %	29.50%	26.50%	36.40%	χ^2^ (2, *N* = 1123) = 11.442; *p* = 0.003
Cardiovascular disease, %	9.80%	8.30%	13.20%	χ^2^ (2, *N* = 1123) = 8.892; *p* = 0.012
Cancer,%	4.40%	3.50%	6.50%	χ^2^ (2, *N* = 1123) = 12.870; *p* = 0.002
COVID-19,%	4.70%	4.70%	4.70%	χ^2^ (2, *N* = 1123) = 0.510; *p* = 0.775
Chronic respiratory disease	12.90%	6.90%	26.70%	χ^2^ (2, *N* = 1123) = 92.156; *p* < 0.001
**Polypharmacy, *n***	2.40 ± 1.76	2.32 ± 1.76	2.60 ± 1.75	*t* (651.755) = -2.445; *p* = 0.015
**Physical activity**				
Daily walking, min/day	11.74 ± 21.68	12.08 ± 20.31	11.21 ± 25.78	*t* (1108) = 0,761; *p* = 0.447
Moderate PA, min/day	15.25 ± 26.13	16.96 ± 26.11	10.42 ± 22.73	*t* (669.271) = 4.025; *p* = 0.000
Vigorous PA, min/day	3.48 ± 9.90	4.05 ± 10.61	2.48 ± 8.54	*t* (721.410) = 2.497; *p* = 0.013
MVPA, min/day	18.70 ± 29.76	21.28 ± 31.02	12.78 ± 25.73	*t* (767.657) = 4.761; *p* < 0.001
Daily sitting time, min/day	325.51 ± 144.40	321.65 ± 146.11	334.45 ± 140.17	*t* (1115) = -1,360; *p* = 0.174
Meeting PA Guidelines,%	20.80%	23.50%	15.00%	χ^2^ (1, *N* = 1117) = 10.247; *p* = 0.001
**Changes in PA during confinement**				
Reduced, %	83.80%	80.2%	92.10%	χ^2^(2, *N* = 1123) = 25.786; *p* < 0.001
Maintained, %	12.90%	16.10%	5.60%	
Augmented, %	3.30%	3.70%	2.30%	
**Changes in daily sitting time**				
Reduced, %	4.20%	4.60%	3.20%	χ^2^ (2, *N* = 1123) = 22.734; *p* < 0.001
Maintained, %	21.90%	25.60%	13.50%	
Augmented, %	73.90%	69.80%	83.30%	
**Depression symptoms, score**	3.57 ± 2.83	2.03 ± 1.31	7.09 ± 2.817	*t* (450.634) = −39.791; *p* < 0.001

*PA, physical activity; MVPA: moderate to vigorous physical activity.*

Table 2 presents hierarchical regression analysis aiming to determine the independent prediction of physical activity variables and sitting time on the depression score. Model 1 (*R*^2^: 0.012; *p* = 0.020) was built considering the variables with clinical/physiological plausibility [age (β = −0.027; 95% CI = −0.042 to 0.016; *p* = 0.383), gender (β = −0.033; 95% CI = −0.912 to 0.253; *p* = 0.267), education level (β = −0.011; 95% CI = −0.332 to 0.230; *p* = 0.723), BMI (β = 0.056; 95% CI = −0.003 to 0.082; *p* = 0.067), and polypharmacy (β = 0.080; 95% CI = 0.031–0.224; *p* = 0.010). The independent variable “daily walking” (β = −0.019; 95% CI = −0.010 to 0.005; *p* = 0.528) within model 2 (*R*^2^: 0.013; *p* = 0.031) did not significantly predict the outcome, increasing 0.1% of the depression score variance. Time spent in moderate physical activity (β = −0.174; 95% CI = −0.026 to −0.012); *p* < 0.001) within model 3 (*R*^2^: 0.039; *p* < 0.001) significantly predicted the depression score, explaining 2.6% of its variance. Model 4 (*R*^2^: 0.042; *p* < 0.001) added time spent in vigorous physical activity (β = −0.054; 95% CI = −0.033 to 0.002; *p* = 0.079), which explained an additional 0.3% of the depression variance. When considering time spent in moderate to vigorous physical activity (β = −0.183; 95% CI = −0.023 to −0.011; *p* < 0.001) together with daily walking and the other covariables, it significantly improved the model, explaining the depression score variance in 2.9% (model 5; *R*^2^: 0.042; *p* < 0.001). Time spent in sitting position did not explain the depression score [models 6 (β sitting time = 0.024; 95% CI = −0.001 to 0.002; *p* = 0.422] and 7 (β sitting time = −0 to 005; 95% CI = −0.001 to 0.001; *p* = 0.876)]. The collinearity between variables was checked for each model, and the VIF values were always below 10. Sensitivity analysis excluding older male adults maintained the same results (Supplementary Material).

**TABLE 2:** Hierarchical linear regression results for physical activity variables as a predictor of symptoms of depression.

		Age	Gender	Education level	BMI	Polypharmacy	Daily walking	Moderate PA	Vigorous PA	MVPA	Sitting time
**Model 1** *R*^2^: 0.012 *F* for change in *R*^2^: 2.701 *p*: 0.020	B	–0.013	–0.330	–0.051	0.040	0.128					
	SE	0.015	0.297	0.143	0.022	0.049					
	β	–0.027	–0.033	–0.011	0.056	0.080					
	95% CI UB; LB	−0.042;0.016	−0.912;0.253	−0.332;0.230	−0.003;0.082	0.031;0.224					
	*p*	0.383	0.267	0.723	0.067	0.010					
**Model 2** *R*^2^: 0.013 *F* for change in *R*^2^: 2.326 *p*: 0.031	B	–0.013	–0.367	–0.042	0.040	0.126	–0.002				
	(SE)	0.015	0.299	0.145	0.022	0.050	0.004				
	β	–0.027	–0.037	–0.009	0.056	0.079	–0.019				
	95% CI UB; LB	−0.042;0.016	−0.954;0.220	−0.326;0.242	−0.003;0.083	0.029;0.224	−0.010;0.005				
	*p*	0.382	0.221	0.773	0.070	0.011	0.528				
**Model 3** *R*^2^: 0.039 *F* for change in *R*^2^: 6.343 *p* < 0.001	B	–0.017	–0.293	–0.046	0.032	0.136	0.005	–0.019			
	(SE)	0.015	0.296	0.143	0.022	0.049	0.004	0.003			
	β	–0.035	–0.030	–0.010	0.045	0.085	0.040	–0.174			
	95% CI UB; LB	−0.045;0.012	−0.873;0.287	−0.327;0.235	−0.011;0.074	0.040;0.232	−0.003;0.013	−0.026;−0.012			
	*p*	0.252	0.322	0.748	0.142	0.006	0.207	0.001			
**Model 4** *R*^2^: 0.042 *F* for change in *R*^2^: 5.947 *p* < 0.001	B	–0.015	–0.313	–0.033	0.033	0.136	0.006	–0.018	–0.015		
	(SE)	0.015	0.296	0.143	0.022	0.049	0.004	0.003	0.009		
	β	–0.032	–0.032	–0.007	0.046	0.085	0.047	–0.166	–0.054		
	95% CI UB; LB	−0.044;0.014	−0.893;0.267	−0.313;0.248	−0.009;0.076	0.040;0.232	−0.002;0.014	−0.025;−0.011	−0.033;0.002		
	*p*	0.304	0.290	0.819	0.126	0.005	0.139	< 0.001	0.079		
**Model 5** *R*^2^: 0.042 *F* for change in *R*^2^: 6.739 *p* < 0.001	B	–0.015	–0.316	–0.030	0.033	0.136	0.006			–0.017	
	(SE)	0.015	0.295	0.143	0.022	0.049	0.004			0.003	
	β	–0.032	–0.032	–0.006	0.047	0.085	0.047			–0.183	
	95% CI UB; LB	−0.044;0.014	−0.895;0.263	−0.311;0.250	−0.009;0.076	0.040;0.232	−0.002;0.014			−0.023;−0.011	
	*p*	0.303	0.285	0.832	0.122	0.005	0.141			< 0.001	
**Model 6** *R*^2^: 0.013 *F* for change in *R*^2^: 2.368 *p*: 0.028	B	–0.016	–0.317	–0.037	0.038	0.129					0.000
	(SE)	0.015	0.297	0.145	0.022	0.049					0.001
	β	–0.033	–0.032	–0.008	0.054	0.081					0.024
	95% CI UB; LB	−0.045;0.013	−0.901;0.266	−0.322;0.248	−0.005;0.081	0.032;0.226					−0.001;0.002
	*p*	0.285	0.286	0.798	0.080	0.009					0.422
**Model 7** *R*^2^: 0.042 *F* for change in *R*^2^: 5.878 *p* < 0.001	B	–0.017	–0.310	–0.041	0.034	0.135	0.006			–0.017	0.000
	(SE)	0.015	0.296	0.145	0.022	0.049	0.004			0.003	0.001
	β	–0.035	–0.031	–0.009	0.047	0.085	0.048			–0.184	–0.005
	95% CI UB; LB	−0.046;0.012	−0.890;0.270	−0.324;0.243	−0.009;0.076	0.039;0.231	−0.002;0.014			−0.023;−0.011	−0.001;0.001
	*p*	0.258	0.295	0.779	0.122	0.006	0.133			< 0.001	0.876

*Non-standardized and standardized beta coefficients are reported. CI, confidence interval; LB, lower bound; UB, upper bond; PA, physical activity; MVPA, moderate to vigorous physical activity. Gender, 0 females, 1 male; Education level, 0 = <6 years; 1 = 6–12 years; 2 = >12 years.*

## Discussion

In this large observational study among previously active older adults, greater time spent in moderate and moderate to vigorous physical activity was associated with a lower depressive score during COVID-19 confinement. To the best of our knowledge, this is the first large community-based study to suggest that time spent in moderate and in moderate to vigorous physical activity during home confinement might impact depressive symptoms of previously active older adults. It was recommended that older adults engage in regular physical activity and avoid sedentary behaviors during confinement; however, 83.80% of older adults who participated in this study diminished their physical activity levels, and this was significantly higher in the participants who reported depressive symptomatology.

Depression is known to be a critical problem among aging populations, often being underdiagnosed and undertreated (Huang et al., 2015). Approximately 20% of older adults who live in the western countries suffer from depression (Volkert et al., 2013). This highly prevalent mental disorder is often related to physical health problems, disability, loss of functionality, and mortality (Cacioppo et al., 2006). The COVID-19 pandemic potentially increases the symptoms of anxiety, depression, and stress (Quittkat et al., 2020). The administrative measures of social isolation, in order to minimize the effects of COVID-19, can cause greater distress and feelings of sadness, especially for those who previously had a large proportion of social contacts in public places (Umberson and Karas Montez, 2010; Armitage and Nellums, 2020). Moreover, it is well known that social isolation and consequent loneliness are considered specific risk factors for depressive symptoms (Cacioppo et al., 2006). Our data showed that being previously active did not prevent 30.40% of the older adults from having depressive symptoms during the COVID-19 pandemic. However, these results, when compared to the prevalence of depression among the Brazilian general population during the COVID-19 pandemic (that was 68%) (Goularte et al., 2021), are much lower and could indicate that previously physically active older adults were somehow protected from depressive symptomatology during the COVID-19 confinement.

The benefits of physical activity in reducing scores of depressive symptoms in older adults are well documented (Kandola et al., 2019). In fact, physical activity and exercise may lower rates of anxiety and depression by triggering the release of endorphin, dopamine, and serotonin, neurotransmitters involved in the body’s stress response, which could help the individuals to remain more stable during extrinsic demanding situations, such as the current pandemic situation (Craft and Perna, 2004). Our results have demonstrated that, from the large spectrum of physical activity intensities, moderate intensity explains 2.6% of the depression score variance, and moderate to vigorous physical activity together explains 2.9%. Conversely, light physical activity (evaluated as walking time) and vigorous physical activity alone were not significantly associated with the depression score. Our results might suggest moderate intensity physical activity as the variable most closely related with lower depression symptomatology. These results might be explained because, with advancing age, in many older adults, the ability to perform some types of physical activity might decrease; thus, moderate exercise intensity may be an especially important component of overall fitness routines. Previous evidence showed that physical activity and exercise have therapeutic effects on individuals’ physical and mental health. For example, Mumba et al. (2020) have showed that recreational moderate and vigorous physical activity was associated with a lower depression score in 2,474 adults and older adults aged 50 years and older. Huang et al. (2015) revealed that exercising at moderate intensity, for 50 min, three times a week, for 12 weeks, was effective in reducing depressive symptoms. In another study with frail and pre-frail older adults, physical exercise was effective in significantly reducing the depression score (Ng et al., 2017). Langoni et al. (2019) developed a 24-weeks intervention with 52 mild cognitively impaired and sedentary older adults. Results demonstrated that the program reduced the median geriatric depression score against the initial value. In addition to reducing depressive symptoms, physical exercise also improves aerobic capacity and the perception of social support, balance, and mobility. These improvements positively affect older adults’ quality of life, diminishing sadness, melancholy, and hopelessness (Huang et al., 2015), maintaining the ability to perform activities of daily living (Demura et al., 2005).

Social isolation, due to home confinement, can lead to reduced everyday physical activity and enlarge sedentary time (Schrempft et al., 2019). Some previous reports have suggested that such restrictions may lead older adults to perform less physical activity compared to the preconfinement period (Jakobsson et al., 2020; Jiménez-Pavón et al., 2020). In fact, our participants reported similar results, as 83.80% of the sample decreased their usual physical activity levels, and 73.90% increased sitting time. When comparing groups with and without depressive symptoms, those with depression symptoms showed a greater reduction in physical activity (92.10% versus 80.20%), as well as a significant increase in sitting time (83.30% versus 69.80%). In agreement, Callow et al. (2020), in a cross-sectional study conducted through an online questionnaire with North American and Canadian older adults, indicated that higher physical activity was associated with lower depressive symptoms during social isolation due to COVID-19. Our results also showed that older adults without depressive symptoms are more likely to meet WHO recommendations for physical activity (23%) compared with 15% among those with depressive symptoms. A reduction of moderate to vigorous physical activity among previously physically active older adults was found, and at least partially, it was a consequence of the interruption of structured physical activities realized by the “Fortaleza Cidade Amiga do Idoso” project. As mentioned by Schrempft et al. (2019), social restrictions and home confinement orders, including the temporary closure of usual activities, such as sports and physical fitness centers, may have compromised physical activity levels.

During the outbreak, the WHO published the new physical activity guidelines, including, for the first time, specific topics on mental health. Indeed, the 2020 WHO physical activity recommendations encourage older adults to exercise between 150 and 300 min per day at moderate to vigorous intensity and to replace sedentary time by any physical activity intensity to sustain physical and mental health (Bull et al., 2020). In this sense, it should be a priority to properly adapt previous in-person interventions to virtual/home-based formats, particularly to those who were physically active before COVID-19. Efficient and safe actions are needed to stimulate the practice of physical activity at home and consequently minimize the psychological effects of social isolation. Moreover, health systems and individual clinicians must be prepared to offer and implement specific physical activity and mental well-being programs in order to reduce the prevalence of depression.

Our analysis had several strengths. A large number of participants, data collection through telephone interviews carried out by acquainted individuals (older adults’ trainers before confinement), and adjustment for a wide range of major risk factors minimized the potential impact of residual confounding. The telephone interview allowed for clear and precise explanations of each question, avoiding misunderstandings.

Potential limitations were also present. The cross-sectional design with a convenience sample makes causal inferences difficult. Results were attained predominantly for women and might not be generalizable to men. However, to overcome this bias, we conducted sensitivity analysis removing males from the procedures, and the results were maintained. Physical activity was assessed with questionnaires that tend to overestimate moderate to vigorous physical activity and underestimate sitting time. Moreover, the percentage of the level of depression explained by physical activity is not very high. This means that other factors may be involved in the changes of the depressive symptoms during the COVID-19 outbreak.

The data suggest that physical activity, particularly the time spent in moderate intensity, might have an important protective role on depressive symptoms during home confinement. Our findings are especially important given the fact that older adults are already more susceptive to depressive symptomatology and confinement might have exacerbated it, so strategies to control or diminish depressive symptoms are of extreme importance. These results support the need for clinicians and policy makers to focus on physical activity interventions through technology and remotely supervise to maintain and promote the mental health of older adults during the COVID-19 pandemic.

## Data Availability Statement

The raw data supporting the conclusions of this article will be made available by the authors, without undue reservation.

## Ethics Statement

The studies involving human participants were reviewed and approved by Núcleo de Produções Culturais e Esportivas (NUPROCE) and the Secretaria de Direitos Humanos e Desenvolvimento Social of Fortaleza, Ceará, Brazil. The patients/participants provided their written informed consent to participate in this study.

## Author Contributions

LB, SG, and ES conceived and designed the analysis. AL, ES, and SG collected data. AL, SG, ES, and LB contributed data and analysis tools. AL, SC, and LB performed the analysis. AL, LB, SC, and LS-M wrote the manuscript. All authors contributed to the article and approved the submitted version.

## Conflict of Interest

The authors declare that the research was conducted in the absence of any commercial or financial relationships that could be construed as a potential conflict of interest.
