# Supplementary material for: Associations Between Depressive Symptoms and Physical Activity Intensity in an Older Adult Population During COVID-19 Lockdown
Source: Front Psychol. 2021 Jun 7;12:644106. doi: 10.3389/fpsyg.2021.644106 (PMC8215341; doi:10.3389/fpsyg.2021.644106)
Supplement: Supplementary file 1 [file Table_1.DOCX]

Supplementary Material

**Supplementary Table.** Sensitivity analysis for multiple linear regression results for physical activity variables as predictor of symptoms of depression excluding older male adults.

|  |  | **Age** | **Education**  **level** | **BMI** | **Polypharmacy** | **Daily**  **walking** | **Moderate**  **PA** | **Vigorous**  **PA** | **MVPA** | **Sitting Time** |
| --- | --- | --- | --- | --- | --- | --- | --- | --- | --- | --- |
| **Model 1**  R^2^: 0.012  F for change in R^2^: 3.083  p: 0.015 | B | -0.012 | -0.034 | 0.044 | 0.137 |  |  |  |  |  |
|  | SE | 0.016 | 0.151 | 0.022 | 0.053 |  |  |  |  |  |
|  | β | -0.025 | -0.007 | 0.062 | 0.084 |  |  |  |  |  |
|  | 95%CI LB; UB | -0.043; 0.019 | -0.331; 0.263 | 0.000; 0.088 | 0.033; 0.040 |  |  |  |  |  |
|  | p | 0.436 | 0.823 | 0.051 | 0.009 |  |  |  |  |  |
| **Model 2**  R^2^: 0.012  F for change in R^2^: 2.488  p: 0.030 | B | -0.012 | -0.022 | 0.044 | 0.133 | -0.002 |  |  |  |  |
|  | (SE) | 0.016 | 0.153 | 0.023 | 0.053 | 0.004 |  |  |  |  |
|  | β | -0.025 | -0.005 | 0.064 | 0.082 | -0.018 |  |  |  |  |
|  | 95%CI LB; UB | -0.043; 0.019 | -0.322; 0.278 | 0.000; 0.090 | 0.029; 0.237 | -0.010; 0.006 |  |  |  |  |
|  | p | 0.438 | 0.886 | 0.048 | 0.012 | 0.562 |  |  |  |  |
| **Model 3**  R^2^: 0.037  F for change in R^2^: 6.313  p<0.001 | B | -0.015 | -0.032 | 0.037 | 0.139 | 0.005 | -0.019 |  |  |  |
|  | (SE) | 0.016 | 0.151 | 0.023 | 0.052 | 0.004 | 0.004 |  |  |  |
|  | β | -0.032 | -0.007 | 0.053 | 0.085 | 0.041 | -0.168 |  |  |  |
|  | 95%CI LB; UB | -0.046; 0.015 | -0.329; 0.264 | -0.007; 0.082 | 0.036; 0.242 | -0.003; 0.014 | -0.026; -0.012 |  |  |  |
|  | p | 0.324 | 0.830 | 0.099 | 0.008 | 0.217 | <0.001 |  |  |  |
| **Model 4**  R^2^: 0.039  F for change in R^2^: 5.768  p<0.001 | B | -0.015 | -0.021 | 0.039 | 0.139 | 0.006 | -0.018 | -0.014 |  |  |
|  | (SE) | 0.016 | 0.151 | 0.023 | 0.052 | 0.004 | 0.004 | 0.009 |  |  |
|  | β | -0.028 | -0.004 | 0.055 | 0.085 | 0.048 | -0.159 | -0.051 |  |  |
|  | 95%CI LB; UB | -0.044; 0.017 | -0.318; 0.276 | -0.006; 0.083 | 0.036; 0.241 | -0.002; 0.015 | -0.025; -0.010 | -0.032; 0.004 |  |  |
|  | p | 0.381 | 0.891 | 0.087 | 0.008 | 0.158 | <0.001 | 0.118 |  |  |
| **Model 5**  R^2^: 0.039  F for change in R^2^: 6.656  p<0.001 | B | -0.014 | -0.017 | 0.039 | 0.138 | 0.006 |  |  | -0.017 |  |
|  | (SE) | 0.016 | 0.151 | 0.023 | 0.052 | 0.004 |  |  | 0.003 |  |
|  | Β | -0.028 | -0.004 | 0.055 | 0.085 | 0.047 |  |  | -0.176 |  |
|  | 95%CI LB; UB | -0.044; 0.017 | -0.314; 0.279 | -0.005; 0.083 | 0.035; 0.241 | -0.002; 0.015 |  |  | -0.024; -0.011 |  |
|  | p | 0.384 | 0.908 | 0.083 | 0.008 | 0.162 |  |  | <0.001 |  |
| **Model 6**  R^2^: 0.013  F for change in R^2^: 2.654  p: 0.022 | B | -0.016 | -0.017 | 0.042 | 0.138 |  |  |  |  | 0.001 |
|  | (SE) | 0.016 | 0.153 | 0.023 | 0.053 |  |  |  |  | 0.001 |
|  | β | -0.032 | -0.004 | 0.059 | 0.085 |  |  |  |  | 0.029 |
|  | 95%CI LB; UB | -0.047; 0.016 | -0.318; 0.284 | -0.003; 0.086 | 0.034 – 0.242 |  |  |  |  | -0.001; 0.002 |
|  | p | 0.328 | 0.911 | 0.065 | 0.009 |  |  |  |  | 0.358 |
| **Model 7**  R^2^: 0.039  F for change in R^2^: 5.684  p<0.001 | B | -0.016 | -0.024 | 0.039 | 0.138 | 0.006 |  |  | -0.017 | 0.000 |
|  | (SE) | 0.016 | 0.153 | 0.023 | 0.053 | 0.004 |  |  | 0.003 | 0.001 |
|  | β | -0.032 | -0.005 | 0.055 | 0.085 | 0.048 |  |  | -0.175 | 0.001 |
|  | 95%CI LB; UB | -0.047; 0.016 | -0.325; 0.276 | -0.005; 0.084 | 0.035; 0.241 | -0.002; 0.015 |  |  | -0.024; -0.011 | -0.001; 0.001 |
|  | p | 0.323 | 0.873 | 0.084 | 0.009 | 0.157 |  |  | <0.001 | 0.973 |
| *Notes*. Non-standardized and standardized beta coefficients are reported. CI: confidence interval; LB: lower bound; UB: upper bond; Gender: 0 females, 1 male; Education level: 0= <6 years; 1= 6 – 12 years; 2= > 12 years, PA: physical activity; MVPA: moderate to vigorous physical activity | | | | | | | | | | |
